# The influence of catheter type, the number of sutures and patients’ age on percutaneous nephrostomy displacement

**DOI:** 10.2478/raon-2025-0037

**Published:** 2025-12-16

**Authors:** Dimitrij Kuhelj, Ana Sustersic, Urban Zdesar

**Affiliations:** 1 Clinical Radiology Institute, University Medical Centre Ljubljana, Ljubljana, Slovenia; 2 Faculty of Medicine, University of Ljubljana, Ljubljana, Slovenia; 3 Faculty of Health Sciences, University of Novo mesto, Novo mesto, Slovenia; 4 Institute of Occupational Safety, Ljubljana, Slovenia

**Keywords:** percutaneous nephrostomy, displacement, catheter type, suture number, age

## Abstract

**Background:**

Percutaneous nephrostomy displacement results in procedure failure, reducing quality of life in patients with hydronephrosis. Scarce data about factors influencing displacement led to evaluation of our data in order to give better insight into this topic.

**Patients and methods:**

Patients admitted for percutaneous nephrostomy (PCN) exchange between March 3^rd^ and October 3^rd^ 2023 were included in our prospective observational study aiming to determine possible factors influencing PCN displacement. Catheter type, number of sutures and patients’ age over 70 years were analyzed. Descriptive statistics and Pearson’s chi-square test were used; value less than 0.05 was determined as statistically significant.

**Results:**

We included 57 patients (35 males; mean age 71.4 years) in the study. Loop catheters with strings were implanted 58 times and without strings 17 times. Fixation was achieved by 55 single and by 20 double sutures. 17 PCN (22.7%) were displaced in designated period. The mean time from PCN implantation to exchange was 4.16 months. Neither catheter type, number of sutures or patients’ age significantly influenced PCN displacement (chi-square 0.57, 0.34 and 0.61, respectively).

**Conclusions:**

No significant difference in PCN displacement between two types of catheters and the number of fixing sutures was detected. Elderly patients had similar rates of PCN displacements as younger ones. The most important causes of PCN displacement remained probably patients’ activity and a care for PCN during months after the implantation. Proper patients’ education and care of the PCN are possibly the keys for long-term success.

## Introduction

Percutaneous nephrostomy (PCN) has been an effective, minimally invasive method of renal collecting system drainage for more than a half of century.^1^ PCN can be temporary or permanent, guided by ultrasound (US), computed tomography (CT), fluoroscopy or combination of methods. Alternatively, catheters may be inserted during renal operations.^2^ Catheter-based drainage is used in malignant or benign obstructions as well as in non-obstructed collective system in case of urinary trauma with high success rates with minimal or even without irradiation of patients.^3^

In patients with prolonged PCN, displacement of catheter is described between 5.5% and 26.3%.^2,4–6^ Catheter displacement leads to obstructive uropathy, infections, electrolytic disbalance and additional hospitalizations, influencing patients’ wellbeing and increasing healthcare costs.

Scarce literature available for the factors influencing PCN displacement, as stated also in recent meta-analysis^7^, as well as a lack of our own data were decisive to review our series of patients. The use of catheter type and the number of sutures, fixing the PCN, is based on the decision of the operator. The aim of our observational study was to determine possible impact of catheter type, the number of sutures fixing PCN to the skin as well as patients’ age on displacement of the PCN at the time of its exchange.

## Patients and methods

Our prospective observational study included consecutive patients, admitted for PCN exchange during 7-month period (March 3rd to October 3rd 2023) in our Institute. Primary procedures were performed by interventional radiologists using US guidance. 18G needle was used for puncture and after fluoroscopic confirmation of pyelocaliceal system with iodine contrast media, 0.035 inches’ guidewire was introduced into renal collective system. Dilator was used to facilitate catheter insertion and PCN catheter was inserted into pyelon through renal parenchyma. Two types of PCN catheters were used, 8 Fr pigtail without locking strings (Optimed Medical Instruments GMBH, Ettlingen, Germany) or 8.5 Fr pigtail with locking strings (Cook Medical, Bloomington, USA). After the PCN catheter insertion it was fixed to skin by single or by two sutures, based on operators’ choice. Patients age above 70 years was also evaluated as a potential factor, influencing PCN displacement.

PCN exchange was performed under fluoroscopic guidance, confirming the PCN position with contrast media. The 0.035 inches’ guidewire was placed through PCN into renal pyelon, the PCN was extracted and exchanged with a new one. In case of displacement, guidewire and/or dilator were inserted through the existing canal if possible, otherwise new, US guided puncture was performed and the procedure was repeated.

PCN exchange was planned in 6 months after the initial procedure, however due to complications in certain patients’ (displacement, obstruction, bleeding etc.) in some patients PCN had to be exchanged earlier.

Descriptive statistics was used to determine percentages and ranges; Pearson’s chi-square test was used to check independence of variables. Value less than 0.05 was determined as statistically significant.

All the patients signed informed consent for a procedure. All procedures performed in present study were in accordance with the ethical standards and with the 1964 Helsinki declaration and its later amendments or comparable ethical standards.

## Results

**FIGURE 1.**
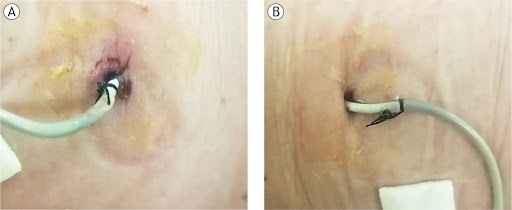
Loop percutaneous nephrostomy (PCN) catheters with strings 6 months after implantation. **(A)** Single suture on the left image is intact, while single suture on the other side **(B)** is not fixed to the skin anymore.

**FIGURE 2.**
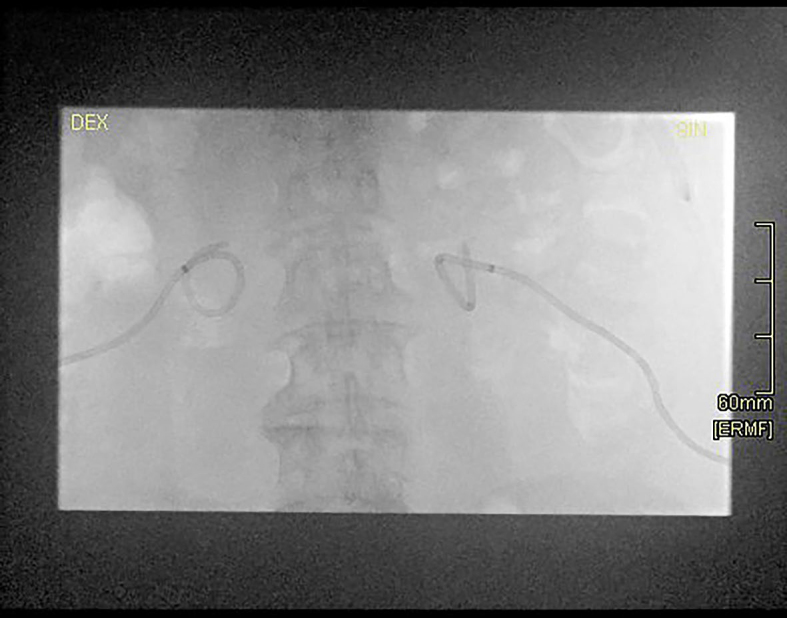
Fluoroscopic image of the same patient. Catheter position is correct, despite non-fixing suture on the right side.

Between March 3rd to October 3rd 2023; 75 PCN were exchanged 62 times in 57 patients. Majority of patients were male (35 males; 22 female) with the mean age 71.4 years (range 43–94). 52 patients were admitted for PNS exchange once, three patients were admitted twice and one was admitted three times, latter always due to PNS displacement. Majority of exchanged PCN catheters were loop catheters with strings (58 PCN; 77.3%), while the remaining 17 were loop catheters without strings. 55 catheters (73.3%) were primarily stabilized by a single suture, while the remaining 20 were stabilized by a double suture. At exchange, sutures were not functional in 24 cases out of 95 sutures made (25.6%) (Figures 1, 2, 3). The mean time from PCN implantation to its exchange was 4.16 months and in 17 pts (24.7%), 17 PCN (22.7%) were displaced at the time of admission.

Out of 58 PCN with strings, 14 (24%) were displaced at the time of admission, while 3 (17.7%) out of 17 without strings were displaced at designated period. The difference between different types of PCN catheters was not statistically significant (chisquare 0.57).

Also, double suturing did not prevent PCN displacement, since in 55 PCN, fixed by a single suture, 14 were displaced (25.4%) and out of 20, fixed by double suture, 3 PCN (15%) were displaced at the time of admission, chi-square 0.34.

Patient age above 70 years did not prove to be significant for PCN displacement, since out of 27 PCN in patients with age less than 70 years, 7 (25.9%) were displaced and in 48 PCN in patients older than 70 years, 10 PCN catheters (20.8%) were displaced (chi-square 0.61).

## Discussion

Catheter displacement is one of the most common complications in long-term derivation of urine by PCN and in the literature it can be as high as 26.3%.^2^ Different types of catheters and skin sutures are used to overcome this problem. The aim of our study was to determine PCN displacement rate by the catheter type (with or without locking strings) and by a number of sutures fixing PCN (one or two sutures).

Perioperatively inserted catheters are often larger and have different shape than minimally invasive percutaneously implanted under imaging guidance, as in our series. Based on that, the procedure itself might result in different ranges of displacement. The frequency of PCN displacement in postoperative and completely percutaneous implantation ranges from 5.5% to 26%.^2,4–6^ However, comparing the rates of catheter displacement in patients with intraoperatively implanted PCN, when large catheters are used (up to 30 Fr) and between PCN catheters, implanted by minimally invasive, image guided procedure, when 8–9 Fr catheters are implanted, might not be objective.

The rate of PCN displacement in our series (22.7%) is higher as in a study by Carrafiello *et al*.^4^, reporting 14.4% of PCN displacement in patients with malignant disease. This study included younger and malignant patients (mean age 65.7 years *vs*. 71.4 years in our series). Anyhow, our data showed that the rate of PCN displacement is not influenced by age (chi-square 0.61). One of the reasons of higher displacement rates in our patients might be the fact that our study included also non-malignant patients, that are more active and as such more prone to inadvertent PCN displacement. More similar rates of PCN displacement to our data were presented by Saad *et al*.^5^, reporting 26% PCN displacement in 6-months period in patients with malignant and benign obstruction.

Two types of catheters are used for PCN in our Institute. Although catheter design with string-locking pig-tail would suggest less displacement, the PCN catheter design showed no statistical difference between both catheter types used in our series (chi-square 0.57). Similar to our data, Chuang *et al*.^8^ find no difference in displacement of catheters with or without strings.

To our knowledge, the number of sutures fixing PCN was not in the focus in any of the studies. Fixing PCN with two sutures is expected to be more durable, however this was not confirmed by our results. Additionally, there was a report of custom-made solution in enhancing PCN fixation to the skin that could reduce PCN displacement.^9^ However, to our knowledge, no data about methods’ success was published since.

We also want to emphasis drawbacks of our study. The main is a relatively low number of patients included as well as observational type of the study. Larger series and randomized data should confirm our results, as stated also by last years’ meta-analysis.^7^ There are also other factors influencing PCN displacement, such as patients’ awareness of the importance of PCN as well as post procedural care of the catheter, that were not taken in consideration in a present study. One of our patients, admitted three times in 7 months for PCN exchange due to its displacement, points towards the importance for post-procedural care of the catheter itself.

PCN displacement is a common complication limiting successful long-term drainage of pyelocaliceal collecting system by PCN inserted during urological operation or by interventional radiologists. It influences patients’ quality of life and increases the costs of the treatment. Our study showed that no kind of catheter is resistant to displacement and that double suturing is no guarantee of solid PCN fixation. Also, in contrast to common belief, elderly patients had similar rates of PCN displacements as younger ones.

The most important causes of PCN displacement remains probably patients’ activity and a care for PCN during days and months after the implantation. Proper patients’ education and explanation about the importance of careful catheter handling during daily activity as well as proper education of nursing staff about the importance of meticulous catheter care after the implantation are probably the keys for long-term effective PCN function. Standard discharge instructions should be a part of discharge protocol for each patient and regular training about catheter care and handling should be provided to nursing staff, including family and other persons, involved in patients’ care.
